# Pigments and Near-Infrared Phosphors Based on Mn^5+^

**DOI:** 10.3390/nano15040275

**Published:** 2025-02-11

**Authors:** Sanja Kuzman, Tatjana Dramićanin, Anatoli I. Popov, Mikhail G. Brik, Miroslav D. Dramićanin

**Affiliations:** 1 Centre of Excellence for Photoconversion, Vinča Institute of Nuclear Sciences—National Institute of the Republic of Serbia, University of Belgrade, 11000 Belgrade, Serbia; tatjana@vinca.rs (T.D.); brik@vinca.rs (M.G.B.); 2 Institute of Solid State Physics, University of Latvia, Kengaraga Street 8, LV-1063 Riga, Latvia; popov@latnet.lv

**Keywords:** phosphors, pigments, transition metals, Mn^5+^, near-infrared emission, luminescence thermometry

## Abstract

The optical properties of Mn^5+^ ions, which are responsible for the intense green–turquoise–blue coloration of Mn^5+^-based pigments and the near-infrared emission of phosphors, are the focus of this article. Mn^5+^ ions enter crystalline matrices in four-fold coordinated positions and can maintain their 5+ valence state when crystalline hosts meet the conditions described in this work. Mn^5+^ ions have [Ar]3d^2^ electronic configuration and always experience a strong crystal field due to a high electric charge; therefore, their lower electronic states have the ^3^A_2_ < ^1^E < ^1^A_1_ < ^3^T_2_ < ^3^T_1_ progression in energy. We present the properties of several Mn^5+^-based pigments and discuss the electronic transitions responsible for their coloration. Specifically, we show that the color is determined by the spin-allowed ^3^A_2_ → ^3^T_1_(^3^F) absorption, which extends across the orange–red–deep red spectral region and is strongly influenced by crystal field strength. The narrow-band emission Mn^5+^-activated near-infrared phosphors arise from the spin-forbidden ^1^E → ^3^A_2_ transition, whose energy is independent of the crystal field strength and determined by the nephelauxetic effect. We demonstrate the linear relationship between ^1^E state energy and the nephelauxetic parameter *β*_1_ using Racah parameter literature data for Mn^5+^ phosphors. Lastly, we address the recent applications of these Mn^5+^ phosphors in luminescence thermometry.

## 1. Introduction

The vivid colors of materials have captivated individuals since antiquity. The principal factor influencing a material’s color is its interaction with light in the visible spectrum (380–750 nm) perceptible to the human eye. Unlike organic dyes, inorganic pigments have superior resistance to heat, light, weathering, solvents, and chemicals, rendering them preferable for artist pigments, exterior coatings, and heat-reflective paints. Inorganic pigments come in a wide variety of colors, from subtle earth tones to vivid blues, greens, and reds. Their colors are highly saturated and fade-resistant, ensuring long-term use in a wide range of applications [1], such as automotive and industrial coatings, paints, plastics, printing inks, cosmetics, and construction materials. Furthermore, pigments play a vital part in the creation of paper, rubber, glass, porcelain, and glazes. Inorganic pigments have high chemical stability, making them resistant to degradation from light, heat, and chemical interactions. This stability makes them ideal for outdoor applications where weather resistance is critical. They also provide a variety of opacity levels, from wholly opaque to transparent, allowing for fine control of visual effects in paints, varnishes, and other materials. Although the fundamental principles of color science for gemstones and minerals are comprehended, precisely predicting the color of inorganic solids continues to be difficult until they are experimentally produced [2].

Pigments are classified based on their different properties. Color is one approach to categorize; white pigments, colorful pigments, and black pigments are the three primary divisions, each with its own set of components. Among white pigments, titanium dioxide, in both rutile and anatase forms [3,4]; zinc sulfide, including lithopones [5]; and zinc oxide take precedence. Colored pigments come in a variety of colors, ranging from blues like complex metal oxides, ultramarine, and Prussian blue [6] to greens like chromium(III) oxide. Yellows include iron(III) oxide hydroxide [7], lead chromate, bismuth vanadate [8], and cadmium sulfide, and reds vary from iron(III) oxide to lanthanum tantalum oxynitride. Carbon black represents the realm of black pigments [9]. Classification based on chemical composition highlights a pigment’s underlying characteristics. Oxides, such as iron oxide and titanium dioxide, are known for their opacity and durability. Sulfides, such as cadmium and zinc sulfide, provide bright colors and resistance to heat. Carbonates, silicates, and hydrated oxides, along with pigments such as zinc carbonate and ultramarine blue, create a flexible palette suitable for a variety of applications. Furthermore, various pigments, such as carbon black and metallic powders, have specialized applications, particularly in the ink and decorative finishes industries.

Transition metal ions are crucial as pigments, offering a broad spectrum of vibrant colors and exceptional stability [10,11]. These ions possess unique properties that make them indispensable in applications such as coatings, plastics, ceramics, and cosmetics. In terms of properties, transition metal pigments exhibit a remarkable variability in color, ranging from deep blues and greens to vivid reds and yellows. Their high opacity and coverage ensure uniform coloration with minimal application, while their chemical stability ensures longevity and resistance to environmental factors like light, heat, and chemicals [12]. Additionally, their compatibility with various binders and substrates facilitates their incorporation into different materials and formulations. Transition metals having a coordination environment with less symmetry and more mixing between the p and d orbitals are more likely to produce bright colors due to the relaxation of selection rules for d–d transitions. Charge transfer transitions typically have high transition probabilities (Laporte permitted), resulting in colorful hues, and they tend to dominate crystal field transition colors when both are present.

Cobalt, copper, iron, nickel, and chrome compounds are commonly used transition metal pigments, each with a unique color and application. Cobalt is known for its brilliant blue and purple hues; copper is prized for its vibrant green and blue shades; iron produces reds, browns, and yellows; nickel provides yellow, green, and gray tones; and chrome, bright yellows and greens. Despite their broad application, these pigments have several drawbacks. Cobalt pigments have been linked to health risks, as cobalt compounds can cause skin sensitivity and respiratory problems in vulnerable persons. Moreover, cobalt pigments’ color may fade or change, especially when exposed to extreme climatic conditions or reactive substances. Copper-based pigments may corrode over time. They can oxidize when exposed to environmental elements such as moisture and oxygen, resulting in color changes and damage to the substance to which they are applied. Iron pigments, while durable and stable, can exhibit a relatively limited color range compared to other pigments. Additionally, they may lack the vibrancy and intensity found in pigments derived from other metals. Moreover, iron-based pigments can be prone to color fading over time, particularly when exposed to harsh environmental conditions such as prolonged sunlight or moisture. Nickel-based pigments pose health risks due to the carcinogenic properties of nickel compounds. Prolonged exposure to nickel pigments, whether through inhalation or skin contact, can lead to allergic reactions and respiratory issues. Additionally, nickel pigments may exhibit limited chemical stability, making them prone to degradation when exposed to certain environmental conditions or chemical agents. Chromium-based pigments raise environmental and health concerns due to the toxicity of chromium compounds. Exposure to chromium pigments can adversely affect human health and the environment, prompting regulatory restrictions and environmental management protocols. Despite these drawbacks, ongoing research and development efforts focus on mitigating the adverse effects of chromium, copper, nickel, and cobalt pigments. Strategies include exploring alternative ions, enhancing production processes, and developing safer substitutes to address environmental and health concerns while maintaining the desired color properties.

One of the solutions is the use of manganese, since it is an essential trace element in the human body and has good biocompatibility. Manganese is known for its ability to stabilize in different oxidation states in crystalline solids, ranging from 1+ to 7+. Each manganese oxidation state has unique electronic and optical properties, which provide opportunities to develop pigments having a wide range of coloration. Well-known examples include manganese brown pigments based on Mn_3_O_4_, manganese violet (NH_4_MnP_2_O_7_), manganese pink (Al_2_O_3_:Mn), orange/red Mn^4+^-activated oxide phosphors [13], black pigments with high reflectivity in the near-infrared spectral range [14], and Mn^5+^-based pigments which are discussed in detail in this paper. Important progress has been achieved recently with the development of blue pigment based on Mn^3+^ optical centers in the hexagonal YInO_3_ and, similarly, in the trigonal bipyramidal sites of hexagonal ScGaZnO_4_, LuGaZnO_4_, and LuGaMgO_4_ [15]. The Mn^3+^ optical centers provide red coloration when introduced into indium sites of the monoclinic Li_3_InB_2_O_6_ [16], purple in YGaO_3_, brown in YAlO_3_ [17] and CaAl_12_O_19_ [18], and violet in LaAlGe_2_O_7_ [19]. Mn^2+^ can facilitate brown coloration when it is introduced to the Zn site in Zn_2_SiO_4_ [20].

Mn^5+^-based pigments are distinguished by their ability to generate vibrant blue and turquoise/green colors [21,22]. Moreover, Mn^5+^-based pigments often exhibit excellent opacity, leading to substantial coverage and consistent coloration in paints, coatings, and other substances. This characteristic is effective for attaining desired aesthetic outcomes with minimal application. Mn^5+^ pigments provide significant stability against deterioration from conditions including light exposure, heat, and chemical interactions. This guarantees the durability and color retention of items containing these pigments, enhancing their lasting attractiveness. Mn^5+^ pigments exhibit compatibility with various binders and substrates, enabling their integration into multiple formulations and materials. This adaptability increases their usefulness across multiple industries and applications. Mn^5+^ pigments play an important part in the creation of eye-catching glazes, tiles, and decorative pottery. Beyond traditional applications, Mn^5+^ pigments may exhibit unique optical and magnetic properties in specialized matrices, offering opportunities for innovation in fields such as electronics, optics, and magnetic materials. These matrices enable the development of novel materials and technologies that leverage the distinctive properties of Mn^5+^ pigments for diverse applications.

Upon activation with low concentrations of Mn^5+^ ions, certain phosphors exhibit light emission in the near-infrared (NIR) spectral range, specifically at wavelengths exceeding 1100 nm and characterized by narrow spectral bands. The narrowband near-infrared luminescence of Mn^5+^ is beneficial for near-infrared lasers [23,24,25] and for narrow-band near-infrared light sources designed for the targeted detection of chemical substances [26]. Recent studies demonstrate that nanoparticles activated by Mn^5+^ function as effective probes for luminescence imaging in deep tissues and luminescence thermometry within the second biological transparency window (1000–1350 nm). Moreover, these nanoparticles demonstrate significant resistance to photochemical degradation [27,28].

This article discusses the electronic processes that regulate the color and emission properties of Mn^5+^ pigments and phosphors, demonstrates the use of crystal field engineering for the control of their color and emission, and reviews the current landscape of Mn^5+^ pigments and phosphors.

## 2. Electronic and Optical Properties of Mn^5+^ Ions

The Mn^5+^ ion has two electrons in the outer 3d electron shell, so its electron configuration is [Ar]3d^2^, where [Ar] denotes the electron configuration of argon with completely filled electron shells 1s^2^2s^2^2p^6^3s^2^3p^6^. Coulomb interaction between two 3d electrons produces 45 degenerated microstates, which are grouped in five LS terms: two spin-triplets—^3^F and ^3^P—and three spin-singlets—^1^D, ^1^G, and ^1^S. Here, the ^2S+1^L notation is used, where S is the total spin, and L is the orbital momentum. According to Hund’s rule, the ^3^F term is the ground state. The degree of degeneracy of these terms is as follows: 21 for ^3^F, 9 for ^3^P, 5 for ^1^D, 9 for ^1^G, and 1 for ^1^S.

The quantitative description of the energy level scheme of a free Mn^5+^ ion requires knowledge of the so-called Racah parameters *B* and *C*, which have dimensions of energy and are linear combinations of the Slater integrals. In terms of the Racah parameters, the energies of the above-given terms are as follows (the energy of the ground term ^3^F is taken as zero): 15*B* for the ^3^P term, 5*B* + 2*C* for the ^1^D term, 12*B* + 2*C* for the ^1^G term, and 22*B* + 7*C* for the ^1^S term. The values of *B* and *C* fitted to the experimentally observed energy levels of free Mn^5+^ ions are *B*_0_ = 1223 cm^−1^ and *C*_0_ = 4613 cm^−1^ [29]. The Hartree–Fock calculated values (which are typically 15–20% overestimated if compared to the experimental values) of the same parameters are *B*_0_ = 1436 cm^−1^ and *C*_0_ = 5450 cm^−1^ [11].

The degenerated energy levels will undergo splitting in the crystal field, with the splitting pattern governed by the point group symmetry of the impurity ion’s site and the magnitude of the splitting reliant on the interionic distances and charges of the adjacent ions. Mn^5+^ ions often occupy four-fold coordinated sites in solids. If such a position has the ideal tetrahedral symmetry described by the T_d_ point group, then the splitting patterns of all above-mentioned LS terms are as follows (the notation of the irreducible representations of the T_d_ group is used): ^3^F → ^3^A_2_ + ^3^T_1_ + ^3^T_2_, ^3^P → ^3^T_1_, ^1^D → ^1^E + ^1^T_2_, ^1^G → ^1^A_1_ + ^1^E + ^1^T_1_ + ^1^T_2_, and ^1^S → ^1^A_1_. If the true symmetry of the Mn^5+^ site is reduced, namely resembling a deformed tetrahedron, the orbitally degenerate states will experience additional splitting, consistent with the symmetry characteristics of the corresponding point group.

The conventional method for analyzing the spectra of transition metal ions in crystals relies on Tanabe–Sugano diagrams [30], which illustrate the splitting of the terms of free ions within cubic crystal fields (in fact, the term “cubic” is a very general one and covers both tetrahedral and octahedral symmetries). These diagrams are usually plotted for a fixed ratio of *C*/*B*; the horizontal axis corresponds to the non-dimensional ratio *Dq*/*B* (*Dq* is the crystal field strength), and the vertical axis is the energy *E* of the crystal field states in terms of the Racah parameters *B* and *E*/*B*.

**Figure 1:**
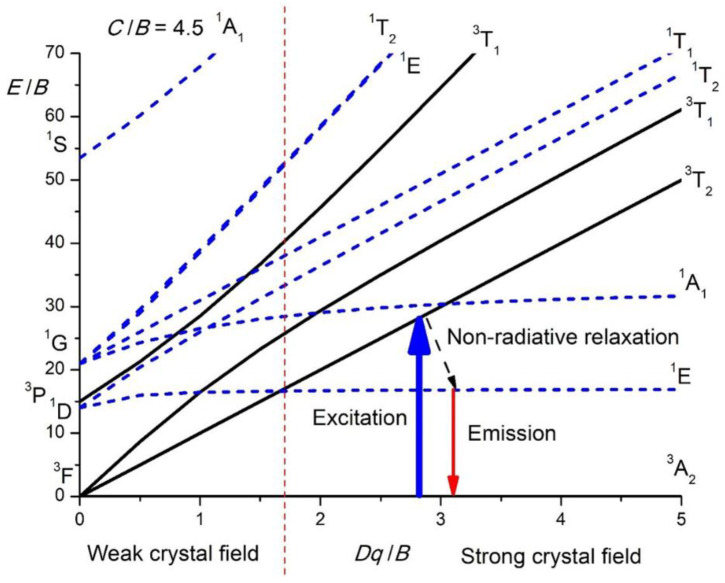
Tanabe–Sugano diagram for the Mn^5+^ ions in the tetrahedral coordination.

Figure 1 shows the Tanabe–Sugano diagram for the 3d^2^ electron configuration in the tetrahedral crystal field (since the Mn^5+^ ions occupy the four-fold coordinated sites). In a majority of crystal field books, the Tanabe–Sugano diagrams are plotted for the octahedral crystal field, but the tetrahedral Tanabe–Sugano diagrams can easily be obtained from the octahedral ones if the conjugate electron configurations d^n^ and d^10-n^ are considered. Thus, the Tanabe–Sugano diagram for Ni^2+^ (3d^8^ configuration) in the octahedral crystal field corresponds to the Tanabe–Sugano diagram for Mn^5+^ or Cr^4+^ (3d^2^ configuration) in the tetrahedral crystal field. The vertical dashed line separates the diagram into two sections designated as the “weak” and “strong” crystal fields. In the former case, the first excited state is the orbital and the spin-triplet ^3^T_2_, and the emission will correspond to the broad spin-allowed ^3^T_2_ → ^3^A_2_ transition. This can be the case with the ions with a smaller electric charge, V^2+^ for example. In the latter case, the first excited state is the orbital doublet and spin-singlet ^1^E, and the emission will be due to the sharp spin-forbidden ^1^E → ^3^A_2_ transition. The Mn^5+^ ions, because of their high electric charge, are always in a strong crystal field situation. Figure 1 also shows a typical way of excitation of the Mn^5+^ emission: at first, the absorption takes place to the ^3^T_2_ state, then the non-radiative relaxation to the ^1^E state occurs, and, finally, the ^1^E → ^3^A_2_ emission transition is realized in the near-infrared spectral range.

A particular feature of the 3d^2^ electron configuration is that the energy difference between the ^3^A_2_ and ^3^T_2_ states coming from the ^3^F term is equal to the 10*Dq*, which gives an easy way of estimating the crystal field strength from the position of the lowest in energy excitation (absorption) peak.

## 3. Spectroscopic Properties, Color, and Photoluminescence of Mn^5+^ in Crystalline Solids

The Mn^5+^ ions tend to enter crystalline matrices in four-fold coordinated positions. From the point of view of charge compensation, the easiest way would be if they substituted for the pentavalent ions, such as P^5+^, V^5+^, or As^5+^. Therefore, the phosphates, vanadates, and arsenates are the most promising materials for doping with the Mn^5+^ ions. With certain charge compensation, it is possible to substitute Mn^5+^ ions for Si^4+^ ions in various silicate hosts [31].

**Figure 2:**
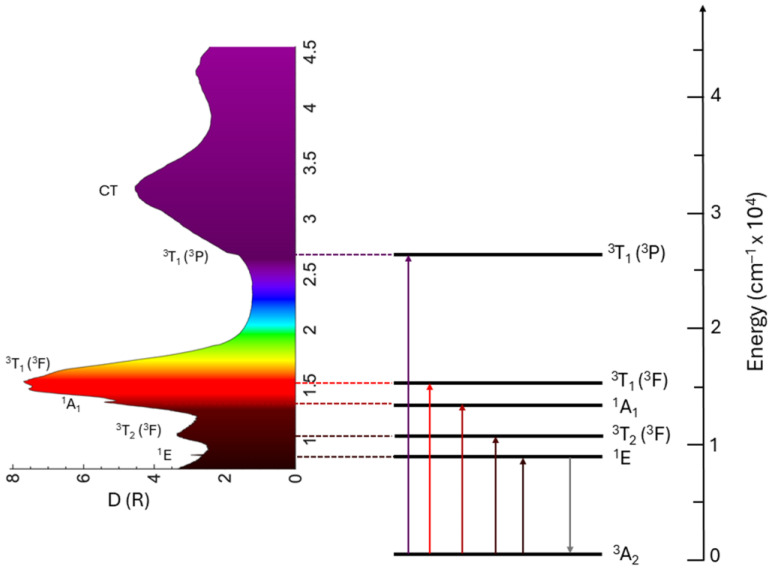
Kubelka–Munk transformation of the diffuse reflectance spectrum of the Ca_6_Ba(PO_4_)_4_O:Mn^5+^ (**left**) and Mn^5+^ energy levels and electronic transitions responsible for color and photoluminescence emission of Ca_6_Ba(PO_4_)_4_O:Mn^5+^ (**right**).

The typical absorption (or excitation) spectrum of Mn^5+^ ions in solids is dominated by two broad structured bands, Figure 2, which are due to the spin-allowed transitions ^3^A_2_ → ^3^T_2_ (^3^F) at about 10,000–12,000 cm^−1^ and ^3^A_2_ → ^3^T_1_ (^3^F) at about 15,000–17,000 cm^−1^, depending on the host. The intensity of the former is usually somewhat lower because, in the T_d_ symmetry, the ^3^A_2_ → ^3^T_2_ transition is forbidden by the group selection rules. The electric dipole-allowed ^3^A_2_ → ^3^T_1_ (^3^P) transition that corresponds to a two-electron jump occurs at higher energies of about 24,000–27,000 cm^−1^. The absorption from the spin-forbidden transitions to the singlet state ^3^A_2_ → ^1^A_1_ (^1^G) occurring around 13,500 cm^−1^ is weak, sharp, and does not depend on the crystal field strength. It is only weakly dependent on the host material’s properties because of the covalent effects (the nephelauxetic effect). The ^3^A_2_ → ^1^E (^1^D) spin-forbidden transition is barely seen in the absorption spectra. Charge transfer bands (CTBs) occur at high energies and are strictly host-dependent.

The color of Mn^5+^ pigments is primarily determined by the ^3^A_2_ → ^3^T_1_ (^3^F) absorption, which extends across the orange–red–deep red spectral region. The energy of the barycenter of this absorption band is significantly influenced by the crystal field strength surrounding Mn^5+^ ions; refer to Figure 1. Therefore, it is possible to tune the pigment color through crystal field engineering, which involves modifying the host crystal structure to alter the distances between Mn^5+^ ions and ligand ions, as well as changing the type of ligand ions. The absorption strength of this transition is influenced by the Mn^5+^ concentration, and any change in this concentration also affects the intensity of pigment color. The CT and ^3^A_2_ → ^3^T_1_ (^3^P) transitions allow absorption in the violet spectral region, with the extent of absorption influenced by the crystal field strength. As a result, the color of the Mn^5+^ pigments is a blend of green and blue, with a slight addition of violet in certain cases.

**Figure 3:**
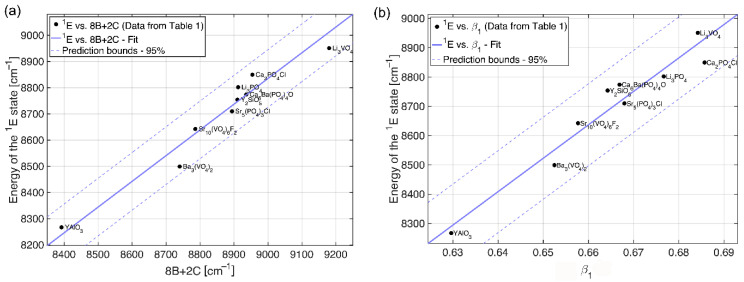
(**a**) The correlation between the energy of the ^1^E state and (8*B* + 2*C*); the full line shows the linear correlation with the slope of 0.9817 ≈ 1, and (**b**) the dependence of the ^1^E state energy on the nephelauxetic parameter *β*_1_ for Mn^5+^ in different hosts listed in Table 1.

The ^1^E (^1^D) → ^3^A_2_ transition determines the emission spectra of the Mn^5+^ ions. It occurs at about 8200–8900 cm^−1^. In certain cases, the weak and broad emission from the ^3^T_2_ (^1^D) → ^3^A_2_ transition can be observed at above-room temperature, usually in systems with low crystal field splitting. The ^1^E emission band is narrow due to weak electron–phonon interaction (the lateral displacement between ^3^A_2_ and ^1^E states is quite small). The ^1^E lifetime is relatively long (half a millisecond) due to the spin-forbidden character of the ^1^E → ^3^A_2_ transition. The Tanabe–Sugano diagram for the 3d^2^ configuration in a tetrahedral crystal field (Figure 1) indicates that the energy difference between the ground state ^3^A_2_ and the first excited state ^1^E is unaffected by the crystal field strength, as both states are parallel to one another. This energy difference closely approximates the energy interval between the ^3^F and ^1^D states of the free ion, defined using the Racah parameters as 8*B* + 2*C*, where *B* and *C* differ among various hosts due to covalent effects. Figure 3a shows a perfect correlation between the energy of the ^1^E state and (8*B* + 2*C*) for Mn^5+^ in different hosts listed in Table 1. Thus, the nephelauxetic effect predominantly influences the energy of the ^1^E state.

For the quantitative analysis of the nephelauxetic effect, a new nephelauxetic parameter *β*_1_ was introduced earlier for the ions with the 3d^3^ electron configuration [32,33] and derived in Ref. [34]:

(1) $$\beta_{1}=\sqrt{{\left(B/B_{0}\right)}^{2}+{\left(C/C_{0}\right)}^{2}}$$

It has been demonstrated in those references that the dependence of energy of the ^2^E→^4^A_2_ spin-forbidden transition for the 3d^3^ electron configuration is a linear function of the *β*_1_ parameter. Later on, the energy of the ^1^E→^3^A_2_ spin-forbidden transition of the Ni^2+^ ions in the octahedral coordination (whose energy level sequence is identical to the case of the Mn^5+^ ions in the tetrahedral coordination, considered in the present work) was also shown to be a linear function of the *β*_1_ parameter. Following that approach, the dependence of the Mn^5+^ ions’ ^1^E state energy on the *β*_1_ parameter is shown in Figure 3b for data taken from Table 1; it appears to be linear (R^2^ of 0.94),

(2) Energy of E1 state cm−1=11418·β1+1101.1.

**Table 1:** Crystal field strength *Dq*, Racah parameters *B* and *C*, nephelauxetic parameter *β*_1_, and the energy of the ^1^E state for the tetrahedrally coordinated Mn^5+^ ions in different crystals.

Host Material	*Dq* [cm^−1^]	*B* [cm^−1^]	*C* [cm^−1^]	*β*_1_	^1^E [cm^−1^]	Reference
Y_2_SiO_5_	1133	550	2255	0.6642	8754.0	[35]
Sr_10_(VO_4_)_6_F_2_	1088	518	2321	0.6577	8642.4	[36]
Sr_5_(PO_4_)_3_Cl	1053	510	2407	0.6679	8710	[37]
Ca_2_PO_4_Cl	1162	455	2657	0.6857	8849.6	[37]
Li_3_PO_4_	1208	475	2556	0.6767	8802.0	[37]
Ca_2_AsO_4_Cl	1030	530	2245	0.6516	N.A. *	[38]
Ca_2_VO_4_Cl	1000	535	2253	0.6557	N.A.	[38]
Li_3_VO_4_	1049	646	2006	0.6842	8950.9	[39]
YAlO_3_	1100	485	2256	0.6296	8267.2	[40]
Ba_3_(VO_4_)_2_	1000	530	2250	0.6525	8499.1	[41]
Ca_6_Ba(PO_4_)_4_O	1060	544	2292	0.669	8773.9	[42]
Ba_2_SiO_4_	1131	419	N.A.	N.A.	8403.4	[43]

* N.A.—not applicable.

It can be noted from the data collected in Table 1 that both Racah parameters *B* and *C* are strongly reduced from their free ion values. This is a clear indication of a high degree of covalency of the Mn^5+^–O^2−^ chemical bonds, which is caused by a high electric charge of the manganese ions pulling the charge density from the oxygen ions towards manganese and thus enhancing the overlap between their wave functions. Higher values of the *β*_1_ parameter are indications of the more ionic nature of the “impurity ion–ligand” chemical bonds, whereas lower *β*_1_ values correspond to enhanced covalent interactions between the impurity ions and ligands.

Oxide complexes including two or more metals represent an important category of thermally stable pigments. The color spectrum of these pigments is generally determined by the presence of various 3d transition metals (V, Cr, Mn, Fe, Co, Ni, or Cu) and a number of coordination polyhedra that can accommodate 3d transition metal ions (tetrahedral, pentahedral, octahedral, or prismatic sites). Phosphate compounds of alkali or alkaline earth metals doped with transition metal ions have been thoroughly examined across many fields, including catalysis [44,45,46], energy research (notably with lithium phosphate cathodes currently under intense study [47,48,49]), and healthcare (where calcium phosphate materials are pivotal biomaterials and continue to be the preferred option for biomedical applications, as substantiated by recent reviews [50,51,52,53]). Moreover, these compounds play a significant role in the coloration of inorganic substances, functioning as dyes or pigments based on their resistance to corrosive environments [54,55,56,57].

Oxide matrices activated in tetrahedral sites by 3d^2^ transition-metal ions, such as Cr^4+^, Mn^5+^, and Fe^6+^, showcase vibrant colors thanks to their significant absorption cross sections in the visible spectral region. Furthermore, these ions exhibit exceptional luminescent characteristics. While Mn^5+^ and Fe^6+^ activated materials display sharp line luminescence in the NIR range [58], compounds activated with Cr^4+^ display a broad emission due to a much weaker nephelauxetic effect. This feature renders them appropriate for use as tunable lasers in the NIR region [59].

Manganese blue, an industrial pigment, is formed by the introduction of Mn^5+^ ions into BaSO_4_. Nonetheless, due to environmental issues, production of this pigment has almost come to a halt. The high price of indium-containing pigments activated by Mn^5+^ has limited their commercial use. To reintroduce Mn^5+^ in commercial pigments, new host material ought to be found [60]. There is a clear need to find cheaper blue pigments with comparable optical characteristics to those currently in use. The main limitation of Mn^5+^ is the need for a host material that can stabilize manganese ions in their 5+ oxidation state. According to Shannon [61], Mn^5+^ exists only in a tetrahedral coordination, with a small effective ionic radius of 33 pm. The (VO_4_)^3−^ group in Ba_3_(VO_4_)_2_ is a good example of a structure for the successful Mn^5+^ ion incorporation at V^5+^ sites (V^5+^ ion radius in tetrahedral coordination is approximately 35.5 pm). Isovalent tetrahedral molecular ions such as (PO_4_)^3−^ or (VO_4_)^3−^ can be easily substituted with (MnO_4_)^3−^ ions, with no charge compensation required because of the same electric charge and similar effective ionic radius (see Table 2). These replacements produce phosphate and vanadate pigments in bright, vivid colors ranging from blue to green [62,63]. These newly developed pigments are easy to synthesize, demonstrate resistance to heat and acids, and are environmentally friendly, offering significant potential for diverse applications where thermal resistance is critical, such as roofing materials. Similar substitutions can be made to (AsO_4_)^3−^ without charge compensation and to (SiO_4_)^2−^ with charge compensation, for example, with Al.

**Figure 4:**
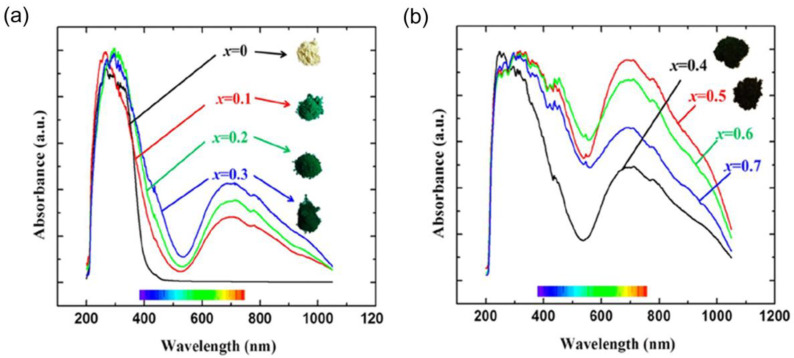
Diffuse reflectance spectra of Ba_2_In_2−x_Mn_x_O_5+x_ samples and their corresponding powder color variations: (**a**) Ba_2_In_2−x_Mn_x_O_5+x_ (x = 0, 0.1, 0.2, 0.3), where the spectrum for x = 0 (Ba_2_In_2_O_5_, white color) is shown for comparison; and (**b**) Ba_2_In_2−x_Mn_x_O_5+x_ (x = 0.4, 0.5, 0.6, 0.7). Reprinted with permission from Ref. [60]. 2013, American Chemical Society.

Several compounds with Mn^5+^ have been documented for their bright coloration. For instance, Mn takes 5+ valence and occupies tetrahedral sites in the brownmillerite-type Ba_2_In_2-x_Mn_x_O_5+x_, providing a light yellow (x = 0), intense turquoise (x = 0.1), green (x = 0.2, 0.3), or dark green (x ≥ 0.4) color [60]. The distinct color of these compounds arises from the substantial absorption of visible light at 500 nm. Under reducing conditions, Mn^5+^ is converted to Mn^3+^, resulting in Ba_2_In_2−x_Mn_x_O_5+x_ turning black. The colors and diffuse reflectance spectra of Ba_2_In_2−x_Mn_x_O_5+x_ phases are shown in Figure 4. The light-yellow appearance of pure Ba_2_In_2_O_5_ results from the band edge tailing into the visible spectrum. Replacing indium with manganese produces a turquoise to green hue due to increased optical absorption in both high- and low-energy parts of the visible spectrum. The high-energy peak predominantly originates from the In–O charge-transfer transitions, whereas a low-energy shoulder results from the Mn–O charge transfer, affecting the visible hue.

The apatite-type structure A_5_(MO_4_)_3_X with A as Ca^2+^, Sr^2+^, Ba^2+^, or Pb^2+^; M as P^5+^, Mn^5+^, Cr^5+^, or V^5+^; and X as F^−^, Cl^−^, OH^−^, provides substantial possibilities for the combining of various A-, M-, and X-site elements, facilitating the creation of compounds with a wide range of advantageous features [64,65]. Notably, apatite-type compounds featuring Mn^5+^ tetrahedrally coordinated by oxygen exhibit promising characteristics as materials for pigments that produce vibrant blue and green colors [66,67].

The 5+ oxidation state of manganese is infrequent in inorganic oxides because of its instability, as Mn^5+^ typically takes more stable oxidation states like 4+ and 7+. However, apatite-type compounds that include Mn^5+^ in tetrahedral coordination at the M-site with alkaline earth elements, especially Ba^2+^, at the A-site exhibit stability because of the high first ionization potential of alkaline earth elements (approximately 5 eV for Ba^2+^), which promotes the preservation of this uncommon oxidation state of Mn [68]. A variety of compounds with an apatite structure have been synthesized, and their structural, optical, and magnetic characteristics and coloration have been studied [69]. Table 3 presents the compounds doped with Mn^5+^ and the synthesis techniques, structural characteristics, and powder color variations resulting from Mn^5+^ ion doping.

**Figure 5:**
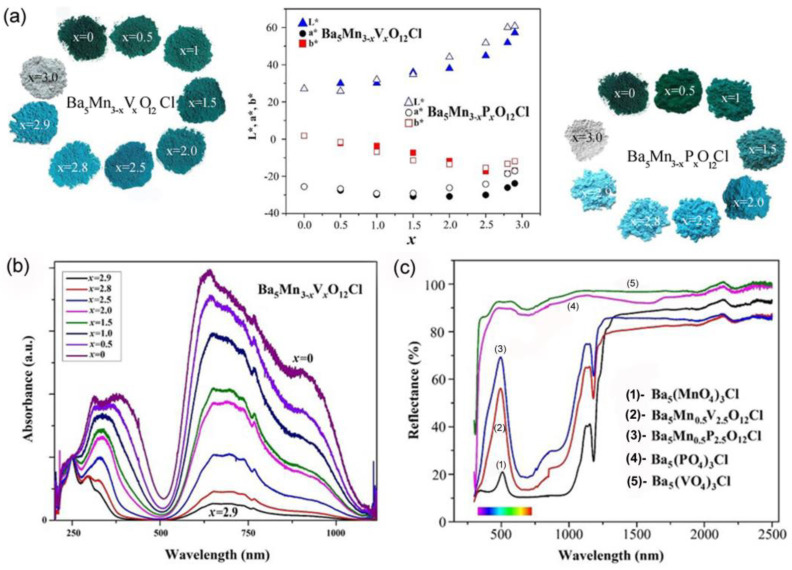
(**a**) L*, a*, b* color parameters of Ba_5_Mn_3-x_V_x_O_12_Cl and Ba_5_Mn_3−x_P_x_O_12_Cl (x = 0; 0.5; 1; 1.5; 2.0; 2.5; 2.8; 2.9; 3.0) samples as a function of the Mn content (x) and color changes with Mn^5+^ doping. (**b**) Diffuse-reflectance spectra of the Ba_5_Mn_3−x_V_x_O_12_Cl series; (**c**) UV-vis and NIR reflectance (%) of Ba_5_Mn_3−x_V_x_O_12_Cl (x = 0, 2.5, 3) and Ba_5_Mn_3−x_P_x_O_12_Cl (x = 0, 2.5, 3) samples as a function of wavelength (nm). Adapted with permission from Ref. [69]. 2016, Elsevier.

The optical properties of Mn^5+^-doped Ba_5_Mn_3-x_M_x_O_12_Cl (M = V, P) apatite structures have been presented by Medina et al. [69]. The doped samples show bright colors from light to dark turquoise and dark green, while undoped Ba_5_(PO_4_)_3_Cl and Ba_5_(VO_4_)_3_Cl are white, Figure 5a. L*, a*, and b* denote the coordinates of the CIELAB color space, with L* indicating lightness, the a*-axis representing the green–red range, and the b*-axis spanning from blue (−b*) to yellow (+b*) [74]. The color coordinates (L*, a*, b*) exhibit an increase in L* and a* values alongside a decrease in b* values with higher Mn content (larger x values) in Ba_5_Mn_3-x_M_x_O_12_Cl (M = V, P) samples (Figure 5). The diffuse reflectance spectra of Ba_5_Mn_3-x_V_x_O_12_Cl pigments, presented in Figure 5b, reveal substantial absorption of red/orange light (around 630 nm) and purple light (around 400 nm), with low absorption in the green/blue spectral region (around 500–520 nm). These optical properties result in the manifestation of green or turquoise colors. Ba_5_V_3_O_12_Cl and Ba_5_P_3_O_12_Cl are white because they do not absorb visible light. The high-energy absorption peak in the UV spectral region is caused by the Mn^5+^–O^2−^ charge transfer. Reduction of manganese concentration decreases absorbance and lightens samples. The authors measured near-infrared reflectance spectra of pigments to assess their cool pigments (Figure 5c). We found out that the reflection intensity decreases with an increase in Mn content, i.e., for pigments of a darker color. Reflectance in the 750–2500 nm range is about 70–85%, making all compounds promising cool pigments.

**Figure 6:**
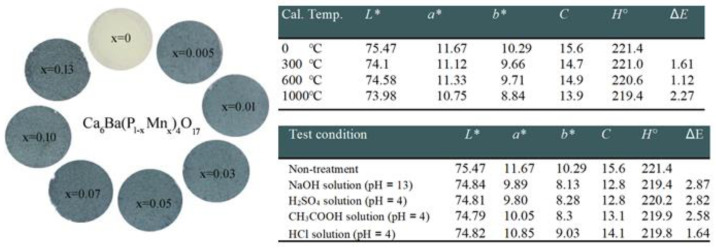
Photographs of the Ca_6_Ba(P_1−x_Mn_x_)_4_O_17_ (0 ≤ x ≤ 0.13) pigments. Tables present color coordination values in the L*, a*, b*, *C, H*⁰, and Δ*E* system of Ca_6_Ba(P_0.99_Mn_0.01_)_4_O_17_ pigments after the thermal stability test and acid and alkali resistance test. Adapted with permission from Ref. [71]. 2017, Elsevier.

Sun Woog Kim et al. [71] analyzed novel inorganic sky-blue pigments, Ca_6_Ba(P_1-x_Mn_x_)_4_O_17_ (0 ≤ x ≤ 0.13), produced using a solid-state reaction technique, emphasizing its chromatic characteristics alongside thermal and chemical stability. The most vivid sky-blue hue, Figure 6, was obtained for Ca_6_Ba(P_0.99_Mn_0.01_)_4_O_17_, which has CIE color parameters of L* = 75.47, a* = 11.67, b* = 10.29, C = 15.6, and H = 221.4 (see the upper table on Figure 6.)

To evaluate the color thermal stability of this pigment, the authors utilized the Ca_6_Ba(P_0.99_Mn_0.01_)_4_O_17_ sample. Firstly, the authors heated the sample at 300 °C, 600 °C, and 1000 °C for 6 h to evaluate the thermal stability of its color performance. Using the equation, the color difference (Δ*E*) of the samples was determined [74] thus:

(3) $$∆E=\sqrt{{∆L}^{*2}+{∆a}^{*2}+{∆b}^{*2}}$$

The samples heated at 300 °C and 600 °C showed color difference (Δ*E*) values of 1.61 and 1.12, respectively, below the minimum difference in color perceptible by humans (Δ*E*_min_ = 2) [75]. The sample heated at 1000 °C had a Δ*E* value of 2.27. The result suggests that the pigment possesses excellent thermal stability within the 0–600 °C region; however, its stability reduces as temperature rises into the 600–1000 °C temperature range. These pigments displayed some chemical color instability in acidic and alkaline solutions; see the upper table in Figure 6. Despite the necessity for enhancements in chemical color stability, this pigment may serve as a promising option for a novel ecologically friendly inorganic sky-blue pigment.

In comparison to Mn^2+^ and Mn^4+^ optical centers, emissions from Mn^5+^ centers have been observed in a much smaller number of host materials. These are, for example, Li_3_PO_4_ [76], Sr_5_(VO_4_)_3_F_4_^,^[25,77], Ba_5_(PO_4_)_3_Cl [78], Sr_5_(PO_4_)_3_Cl [78,79,80], Ca_2_PO_4_Cl [78,79], Ca_2_VO_4_Cl [78,79], Sr_2_VO_4_Cl [78,79], Ca_6_Ba(PO_4_)_4_O [42], Y_2_SiO_5_ [31], and M_2_SiO_4_ (M  =  Ba, Sr, Ca) [43].

The early application of Mn^5+^ emission has been concentrated on the development of NIR-emitting solid-state lasers [23,25]. The laser action is characterized using a three-level laser scheme, and it has been demonstrated that the long lifetime of the ^1^E state, together with its substantial visible absorption, makes the Mn^5+^ system suitable for flashlamp pumping. However, the Mn^5+^ laser’s tunability is severely constrained due to its narrow emission. The typical internal quantum efficiencies (IQE) of Mn^5+^-activated phosphors vary from 20% to 40%, with new findings indicating a potential enhancement in IQE with Bi^3+^ co-doping [81].

## 4. Luminescence Thermometry with Mn^5+^ Ions

Mn^5+^ has the benefit of activating nanoparticles, making them ideal probes for luminescent imaging in deep tissues and luminescent thermometry in the second window of biological transparency (1000–1350 nm). Recently, Piotrowski et al. [82] demonstrated Mn^5+^ lifetime-based thermal imaging in the optical transparency windows through skin-mimicking tissue phantoms. Dramicanin et al. [42] presented and explained the near-infrared luminescence of Ca_6_Ba(PO_4_)_4_O:Mn^5+^ and demonstrated its use for temperature sensing in the near-infrared spectral region. When excited within a broad and strong absorption band spanning from 500 to 1000 nm, this phosphor provides an ultra-narrow emission (FWHM = 5 nm) centered at 1140 nm, originating from the spin-forbidden ^1^E→^3^A_2_ transition. They discovered that the ^1^*E* emission is quenched due to thermally assisted crossover with the ^3^T_2_ state and that the relatively high Debye temperature of 783 K for Ca_6_Ba(PO_4_)_4_O facilitates efficient emission. A high Debye temperature indicates the material’s rigid structure. The increased structural rigidity restricts the non-radiative transition of photons, resulting in enhanced quantum efficiency. This phosphor has been effective in luminescence intensity ratio thermometry, with a relative sensitivity of 1.92% K^−1^ and temperature resolution of 0.2 K within the physiological temperature range. The relative sensitivity of a thermometer is defined as the rate of the temperature-induced change in a measured luminescence feature divided by the magnitude of this feature, and the temperature resolution is the smallest change in a temperature that causes a perceptible change in the measured luminescence feature [83]. While there are no established thresholds at which the materials are considered promising for thermometers since different applications require different measurement performances and temperature operating ranges, it is generally assumed that thermometers with relative sensitivities exceeding 1% K^−1^ and temperature resolution better than 0.5 K around room temperature may be considered promising [84,85].

**Figure 7:**
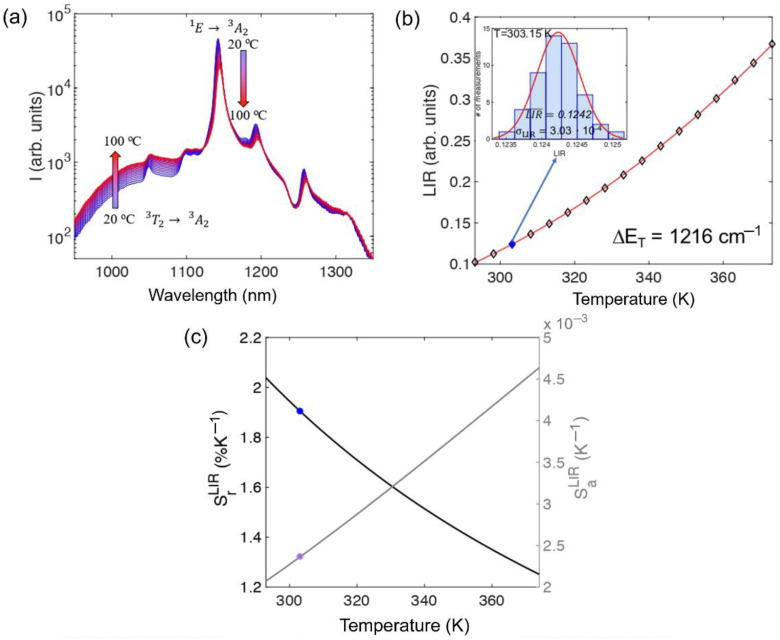
(**a**) Photoluminescence emission spectra of Ca_6_Ba(PO_4_)_4_O:Mn^5+^ powder measured at different temperatures; (**b**) luminescence intensity ratio (LIR) as a function of temperature (experimental data—diamond markers). The insert shows the LIR distribution histogram measured at 303.15 K (30 °C)—filled diamond marker; (**c**) calculated absolute and relative sensitivities (marked values at 303.15 K (30 °C)). Reprinted from Ref. [42].

As illustrated in Figure 7a, an increase in temperature results in the enhancement of the broad emission peak from the ^3^A_2_ level within the 950 nm to 1030 nm range, while the narrow emission peak from the ^1^E level around 1140 nm decreases. This phenomenon occurs due to thermalization between the ^1^E and ^3^T_2_ levels, with the energy difference (ΔE_*T*_ = 1216 cm^−1^) being overcome by thermally excited electrons. To experimentally determine the uncertainty in the luminescence intensity ratio (LIR), 50 emission spectra were recorded at each temperature. The mean of these measurements was used as the LIR value, and the standard deviation (σ_*LIR*_) was considered the uncertainty in LIR, as depicted in the inset of Figure 7b (distribution measured at 30 °C). Figure 5c shows that the relative sensitivity value, represented by blue dots for measurements taken at 30 °C, ranges from 2.35%K^−1^ to 1.26%K^−1^ over the temperature range, with a value of 1.92% K^−1^ at 30 °C. This sensitivity is among the highest recorded for luminescent thermometers operating within the second biological transparency window.

## 5. Conclusions

The color of Mn^5+^ pigments is influenced by the spectral positions and intensity of absorptions from the spin-allowed ^3^A_2_ → ^3^T_1_ (^3^F) (the orange–deep red spectral region) and ^3^A_2_ → ^3^T_1_ (^3^P) (the violet spectral region) electronic transitions. The former can be controlled by engineering the crystal field surrounding the Mn^5+^ ions (by varying manganese-ligand bond lengths, bond angles, and ligand type), whereas the latter can be altered by changing Mn^5+^ concentration. The charge transfer between Mn^5+^ and ligands also has an effect on violet absorption. The turquoise-blue coloring of the Mn^5+^ pigments is a result of these absorptions. The narrow-band near-infrared emission with a long lifetime, typically around a half millisecond, occurs in low-doped materials. It is composed of emission from the spin-forbidden ^1^E → ^3^A_2_ transition and its vibrational sidebands. The energy of the ^1^E emission is strongly influenced by the nephelauxetic effect (covalency) and is not dependent on the crystal field strength. We showed here that the energy of the ^1^E state is linearly dependent on the value of the nephelauxetic parameter *β*_1_. The weak and broad emission from the spin-allowed ^3^T_2_ (^1^D) → ^3^A_2_ transition can be observed at temperatures above room temperature. This phenomenon is typically observed in systems with small crystal field splitting when the energy difference between ^1^E and ^3^T_2_ is sufficiently small to allow thermalization. Despite the difficulty of maintaining manganese’s 5+ valence state when added into solids, the number of reported Mn^5+^ pigments and phosphors is growing over time. Suitable hosts for Mn^5+^ doping typically consist of (PO_4_)^3−^, (VO_4_)^3−^, or (SiO_4_)^2−^ groups, where Mn^5+^ replaces P^5+^, V^5+^, or Si^4+^ in tetrahedral coordination, and contain electropositive alkaline earth metals such as Ba, Sr, and Ca. Mn^5+^ pigments are generally non-toxic and possess favorable stability, which presents a significant opportunity for their future application. Their narrow-band near-infrared emission was initially evaluated for solid-state laser development but has recently been applied in bioimaging and biothermal imaging within the second biological transparency window. Luminescence thermometry sensors utilizing Mn^5+^ emission typically exhibit a large relative sensitivity of approximately 2% K⁻¹ at room temperature. This, combined with small uncertainties in the measurements of intense ^1^E emissions, results in high precision and accuracy, achieving around 0.2 K in measurements.

## Figures and Tables

**Table 2:** Effective ionic radii in tetrahedral coordination of selected metals, according to Shannon [57].

Ion	Mn^5+^	V^5+^	P^5+^	As^5+^	Si^4+^
Radius	33 pm	35.5 pm	17 pm	33.5 pm	26 pm

**Table 3:** Synthesized materials doped with Mn^5+^ used as pigments.

Material	Synthesis	Structure	Color	Reference
Ba_5_Mn_3−x_M_x_O_12_Cl (M = V^5+^, P^5+^) (x = 0–3.0)	Solid-state Sol-gel process	hexagonal P6_3_/m	White (x = 3.0) Turquoise (x = 1.5) Dark green (x = 0)	[69]
Ba_3_(V_1−x_Mn_x_O_4_)_2_ (0 < x ≤ 1.0)	Conventional ceramic route	hexagonal R3m	Turquoise (x = 0.10) Green (x = 0.20, 0.25) Dark green (x ≥ 0.50)	[70]
Ba_2_Ti_1−x_Mn_x_O_4+x/2_ (0 < x ≤ 0.15) Ba_2_Si_1−x_Mn_x_O_4_ x = 0.10 and 0.15	Conventional ceramic route	Ba_2_Ti_1−x_Mn_x_O_4+x/2_ orthorhombic P2_1_nb Ba_2_Si_1−x_Mn_x_O_4_ orthorhombic Pmcn	Turquoise (x = 0.10, 0.15) Turquoise (x = 0.10, 0.15)	[70]
Ba_3_(P_1−x_Mn_x_O_4_)_2_ x = 0, 0.02, 0.05, 0.10, 0.25, 0.30, 0.40, 0.50, 0.75, 0.90, 1.00	Conventional ceramic route	hexagonal R3m	Sky blue (x = 0.02–0.10) Turquoise blue (x = 0.25–0.40) Green and dark green (x ≥ 0.50)	[54]
Ca_6_Ba(P_1−x_Mn_x_)_4_O_17_ (0.005 ≤ x ≤ 0.13)	Solid-state	monoclinic C2/m (No. 12)	Sky blue color near the green	[71]
BaAl_2−x_Mn_x_O_4+y_ (0 ≤ x ≤ 0.10)	Sol-gel process	ferroelectric phase P6_3_ when T increase paraelectric phase P6_3_22	White (x = 0.05) Green blue (0.01 ≤ x ≤ 0.03) Green yellow (0.04 ≤ x ≤ 0.10)	[22]
Ba_7_Al_2−x_Mn_x_O_10+y_ (0 ≤ x ≤ 0.7)	Solid-state		From cyan to ocean blue	[72]
Ba_2_In_2−x_Mn_x_O_5+x_ (x = 0.1−0.7)	Solid-state	Ba_2_In_1.9_Mn_0.2_O_5.1_ Orthorhombic Icmm Ba_2_In_1.8_Mn_0.2_O_5.2_ tetragonal I4/mcm Ba_2_In_2−x_Mn_x_O_5+x_ (x = 0.4, 0.5, 0.6) cubic phases Pm3m	Light yellow (x = 0) Turquoise (x = 0.1) Green (0.2, 0.3) Dark green (x ≥ 0.4)	[60]
Ba_10_(P_1−x_Mn_x_O_4_)_6_F_2_ x = (0.1–1)	Solid-state	hexagonal P6_3_/m	Turquoise blue to dark green	[73]

## Data Availability

The data presented in this study are available on request from the corresponding author.
